# Bile reinfusion is associated with shifts in host-microbiota metabolic profiles following cholangiocarcinoma-associated microbiota transplantation

**DOI:** 10.1038/s41598-026-58698-2

**Published:** 2026-06-26

**Authors:** Thanaporn Saensupha, Anyarin Jirahiranpat, Natthawut Pratumwan, Theerayut Bubpamala, Wassana Jamnongkan, Thanaporn Kulthawatsiri, Manida Suksawat, Kanokrat Thaiwatcharamas, Waraporn Auiewiriyanukul, Marisa Attapong, Navaporn Sritong, Benjamin H. Mullish, Jutarop Phetcharaburanin

**Affiliations:** 1 Medical Biochemistry and Molecular Biology Graduate Study Program, Faculty of Medicine, Khon Kaen University, Khon Kaen, Thailand; 2 The National Phenome Institute, Office of the President, Khon Kaen University, Khon Kaen, Thailand; 3 Department of Systems Biosciences and Computational Medicine, Faculty of Medicine, Khon Kaen University, Khon Kaen, Thailand; 4 International College, Khon Kaen University, Khon Kaen, Thailand; 5 Department of Surgery, Faculty of Medicine, Khon Kaen University, Khon Kaen, Thailand; 6 Department of Biochemistry, Faculty of Science, Kasetsart University, Bangkok, Thailand; 7 Division of Digestive Diseases, Department of Metabolism, Digestion and Reproduction, Faculty of Medicine, Imperial College London, London, UK; 8 Department of Hepatology, St Mary’s Hospital, Imperial College Healthcare NHS Trust, London, UK

**Keywords:** Biochemistry, Metabolomics, Liver cancer

## Abstract

Cholangiocarcinoma (CCA) is a heterogeneous group of malignant tumours originating along the biliary tract. Previous studies have demonstrated that CCA is characterised by altered gut microbial composition and disrupted bile acid metabolism, both of which are critical determinants of host metabolic homeostasis. Although bile reinfusion (BR) has been proposed to improve surgical outcomes in CCA patients, its systemic metabolic effects and interaction with gut microbiota remain poorly understood. Here, we employed faecal microbiota transplantation (FMT) from CCA patients, with or without BR, into Wistar rats to investigate host-microbiota metabolic interactions using integrated ^1^H NMR-based metabolomics and full-length 16S rRNA gene sequencing. Rats receiving CCA-derived microbiota displayed altered systemic metabolic phenotypes, characterised by lower levels of glucose, lactate, and succinate compared to normal microbiota recipients, whilst no significant differences in faecal metabolites were observed between these groups. Notably, BR was associated with shifts in gut microbial composition, marked by enrichment of Lactobacillaceae, altered intestinal fermentation metabolites (decreased short-chain fatty acids and increased succinate), and a convergence of peripheral plasma metabolite profiles towards those observed in healthy microbiota recipients. These findings reveal associations between bile reinfusion and shifts in microbial composition and systemic metabolic phenotypes, providing a basis for investigating microbiota-bile acid-host metabolic crosstalk and potential therapeutic implications for managing CCA-associated dysbiosis.

**Supplementary Information:**

The online version contains supplementary material available at https://doi.org/10.1038/s41598-026-58698-2.

## Introduction

Cholangiocarcinoma (CCA), also known as bile duct cancer, is a malignant tumour of epithelial cells that can arise anywhere in the biliary tract. It represents 10% − 20% of all hepatic cancers and is the second most prevalent primary liver malignancy, behind hepatocellular carcinoma (HCC)^1^. CCA is still a major public health problem, particularly in Northeast of Thailand and the neighbouring countries e.g. Lao, Vietnam, and Cambodia. Moreover, CCA is typically asymptomatic at the early stage and diagnosed at the advanced stage, severely limiting treatment options and leading to poor prognosis^2^. Consequently, development of novel strategies for CCA diagnosis and treatment remains crucial.

The gut microbiota is a complex community of microorganisms residing throughout the mammalian gastrointestinal tract. These microbial communities, comprising bacteria, viruses, and some eukaryotes, establish themselves in the gastrointestinal tract soon after birth and contribute substantially to host health^3^. Host metabolic pathways are regulated by the gut microbiota through interactive host-microbiota metabolic signalling and immune-inflammatory axes that physiologically link the gut, liver, muscle, and brain^4^. Alterations in gut microbiota composition and function – commonly referred to as dysbiosis – have been associated with disruptions in microbial and host-microbial co-metabolism, and with a wide range of disease states including CCA^5–8^. Many studies showed the association between gut microbiota dysbiosis and host-microbial metabolites changes in CCA^7–9^. It is important to note, however, that current evidence is primarily correlational and that causal relationships between specific microbial alterations and clinical disease states remain to be established. Studying the associations between gut microbiota and host metabolic profiles in CCA may reveal novel aspects potentially meaningful for clinical management.

Bile acids represent one class of metabolites with particular relevance to CCA. Bile acids are synthesised in the liver from cholesterol and undergo microbial biotransformation in the intestine to yield secondary bile acids^9^. Bile acids can influence gut microbiota composition both through direct antimicrobial properties and indirectly through modulation of host signalling pathways; it is recognised, however, that dietary factors – which are the primary determinants of bile acid secretion patterns – are important upstream drivers of bile acid–microbiota interactions^10,11^. Together, the gut microbiota and bile acid pool form a reciprocally interactive axis in which each modulates the composition and activity of the other. For CCA patients, preoperative biliary drainage has been suggested to enhance liver function prior to surgery and reduce postoperative complications^12^. External biliary drainage, however, disrupts the enterohepatic circulation of bile. Bile reinfusion (BR) has been proposed to restore this physiological process, with potential benefits for intestinal mucosal barrier integrity and microbial homeostasis^13–15^.

The gut microbiota may serve as a functional transducer of bile acid-mediated signals: bile acids reshape microbial community structure and activity, which in turn alters the profile of host-microbial co-metabolites reaching the portal and systemic circulations. Understanding this bile acid–microbiota–host metabolic axis could, therefore, identify the microbiota as a tractable target for interventions aimed at improving metabolic homeostasis in CCA patients. Currently, mechanistic data on the systemic metabolic consequences of CCA-associated microbiota and the effects of BR on this system remain limited. In this study, we established an *in vivo* CCA-derived FMT rat model and employed ¹H NMR-based metabolomics and full-length 16S rRNA gene sequencing to characterise associations between CCA-derived microbiota transplantation and bile reinfusion and the host metabolic phenotype. Understanding such associations may offer novel prospects for developing monitoring and treatment strategies for CCA patients.

## Results

### Animal body weight, and overview of metabolic profiles, following FMT and bile reinfusion

Body weight was recorded at each sampling time point (day − 6, 0, 1, 8, and 14) to monitor the general health and welfare of the animals throughout the experiment (Fig. S1). All groups showed a comparable body weight trajectory over the study period, with no statistically significant differences between control (Ct), normal (Nr), CCA (Cr), and CCA with bile (CrB) groups at any individual time point, as assessed by two-way ANOVA (Fig. S1). A transient reduction in body weight was observed across all groups following antibiotic administration and FMT at day 0–1, consistent with the expected effects of antibiotic depletion of the intestinal microbiota and the gavage procedure. Body weights recovered progressively thereafter in all groups and were not significantly different at day 14. These findings indicate that neither CCA-associated microbiota transplantation nor bile reinfusion adversely affected overall animal welfare or produced overt systemic toxicity over the 14-day study period, and that any observed metabolic differences between groups cannot be attributed to differences in body weight.

**Fig. 1:**
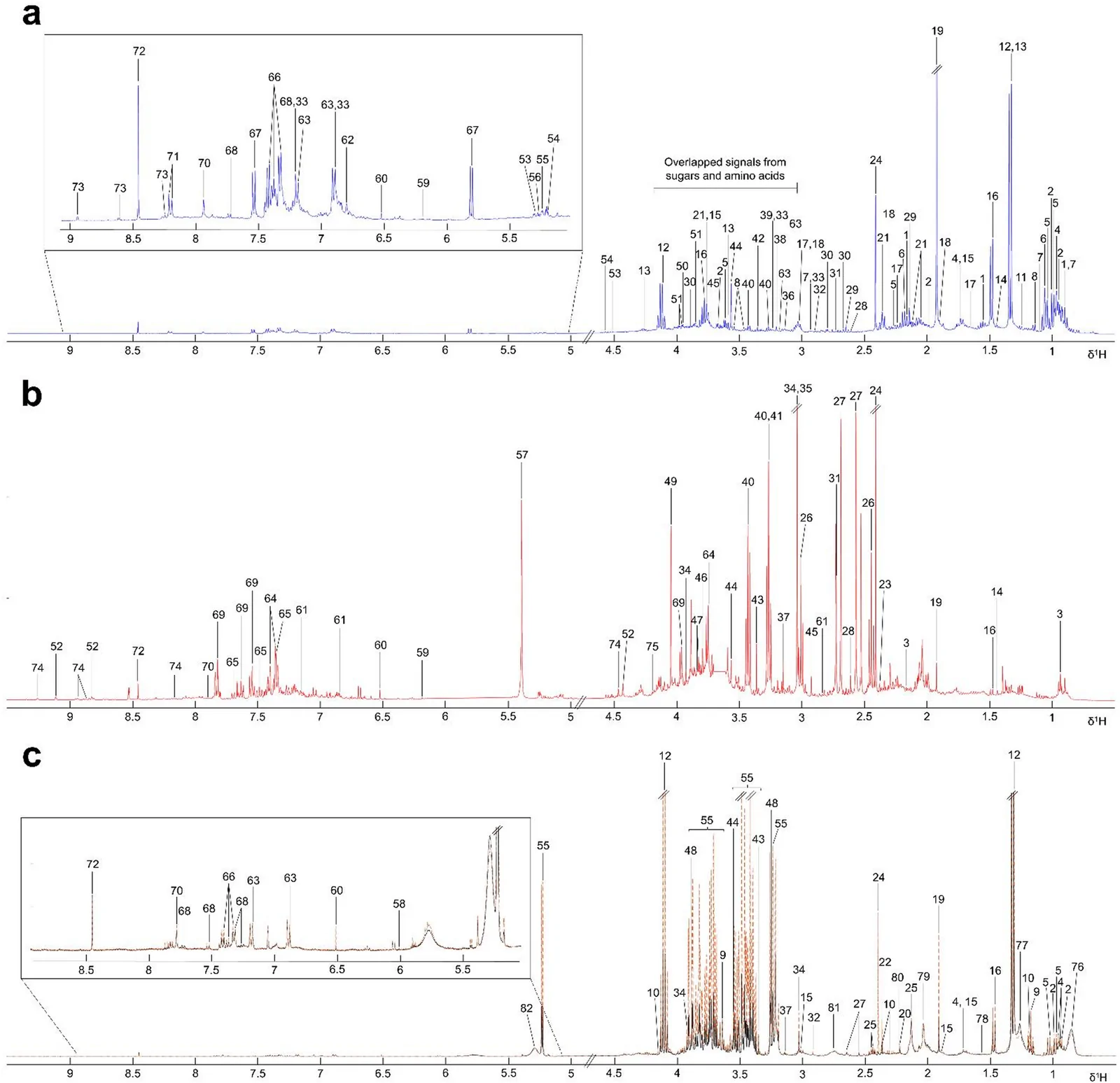
¹H NMR median spectra of biofluids. ¹H NMR median spectra of (**a**) faeces, (**b**) urine, (**c**) plasma samples obtained from control (Ct), normal (Nr), CCA (Cr), and CCA with bile (CrB) groups of Wistar rats at baseline, day 0, day 1, day 8, and day 14. Key metabolite regions are annotated for principal faecal SCFAs (acetate, propionate, butyrate), succinate, and principal plasma metabolites (glucose, lactate). For median spectra of plasma samples, the black solid line represents peripheral plasma and the orange dashed line represents portal vein plasma.

**Fig. 2:**
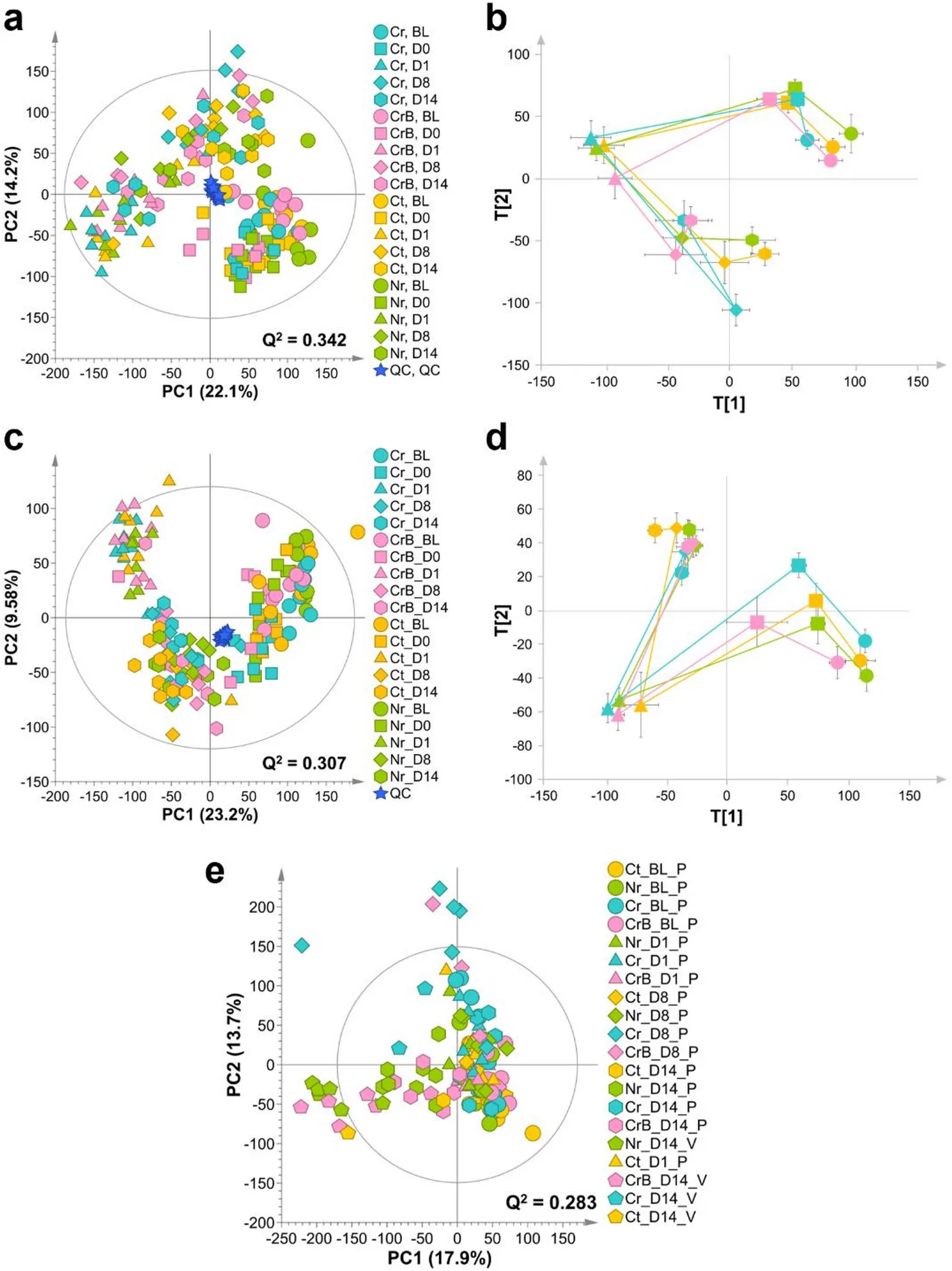
Principal component analysis of faecal, urinary, and plasma metabolome data. PCA scores plot of faecal (**a**,** b**), urinary (**c**,** d**), and plasma (**e**) metabolic profiles obtained from Ct (yellow), Nr (green), Cr (blue) and CrB (pink) Wistar rats at day − 6 (circle), day 0 (square), day 1 (triangle-up), day 8 (diamond), day 14 (hexagon), and QC samples (star). (**a**,** c**) PCA scores plots confirming absence of batch effects based on QC clustering; percentage variance explained by each PC and Q² are indicated. (**b**,** d**) Time-trajectory PCA scores plots showing group mean PC1 vs. PC2 ± SE at each time point.

Faecal, urinary and plasma metabolites were characterised using ¹H NMR spectroscopy (Fig. 1; Table S1). To provide an overview of the metabolic dataset structure, principal component analysis (PCA) was performed on faecal, urinary, and plasma ¹H NMR spectral data from Ct, Nr, Cr, and CrB groups at all time points. Tight clustering of quality control (QC) samples in PCA scores plots confirmed the absence of significant batch effects across the analytical runs (Fig. 2a, faecal: R² = PC1 22.1%, PC2 14.2%, Q² = 0.342; Fig. 2c, urinary: R² = PC1 23.2%, PC2 9.58%, Q² = 0.307). Time-trajectory PCA scores plots demonstrated a time-dependent shift in metabolic profiles from day − 6 to day 14 in all groups following antibiotic depletion and FMT, reflecting the expected dynamics of microbiota engraftment and maturation (Fig. 2b, d). This time-dependent shift was observed across all experimental groups and is distinct from between-group differences, which are addressed separately below using pairwise OPLS-DA analyses with univariate validation. PCA-based PERMANOVA further confirmed statistically significant overall metabolic profile separation between experimental groups across the study period in both faeces and plasma (Fig. S2, *p* < 0.05). PCA of plasma metabolic profiles (Fig. 2e) demonstrated clear separation of portal vein plasma from peripheral plasma along PC1, confirming that these compartments exhibit distinct metabolic compositions and were analysed accordingly.

### Faecal metabolic alterations following FMT and bile reinfusion

Faecal metabolites were identified by ¹H NMR spectroscopy (Fig. 1a; Table S1). To identify specific faecal metabolic differences between groups, pairwise OPLS-DA analyses were conducted at day 1, day 8, and day 14 (Table S2). All OPLS-DA models were validated by permutation testing (999 permutations, *p* < 0.05), and significant metabolites were confirmed by Kruskal-Wallis test with Benjamini-Hochberg correction.

**Fig. 3:**
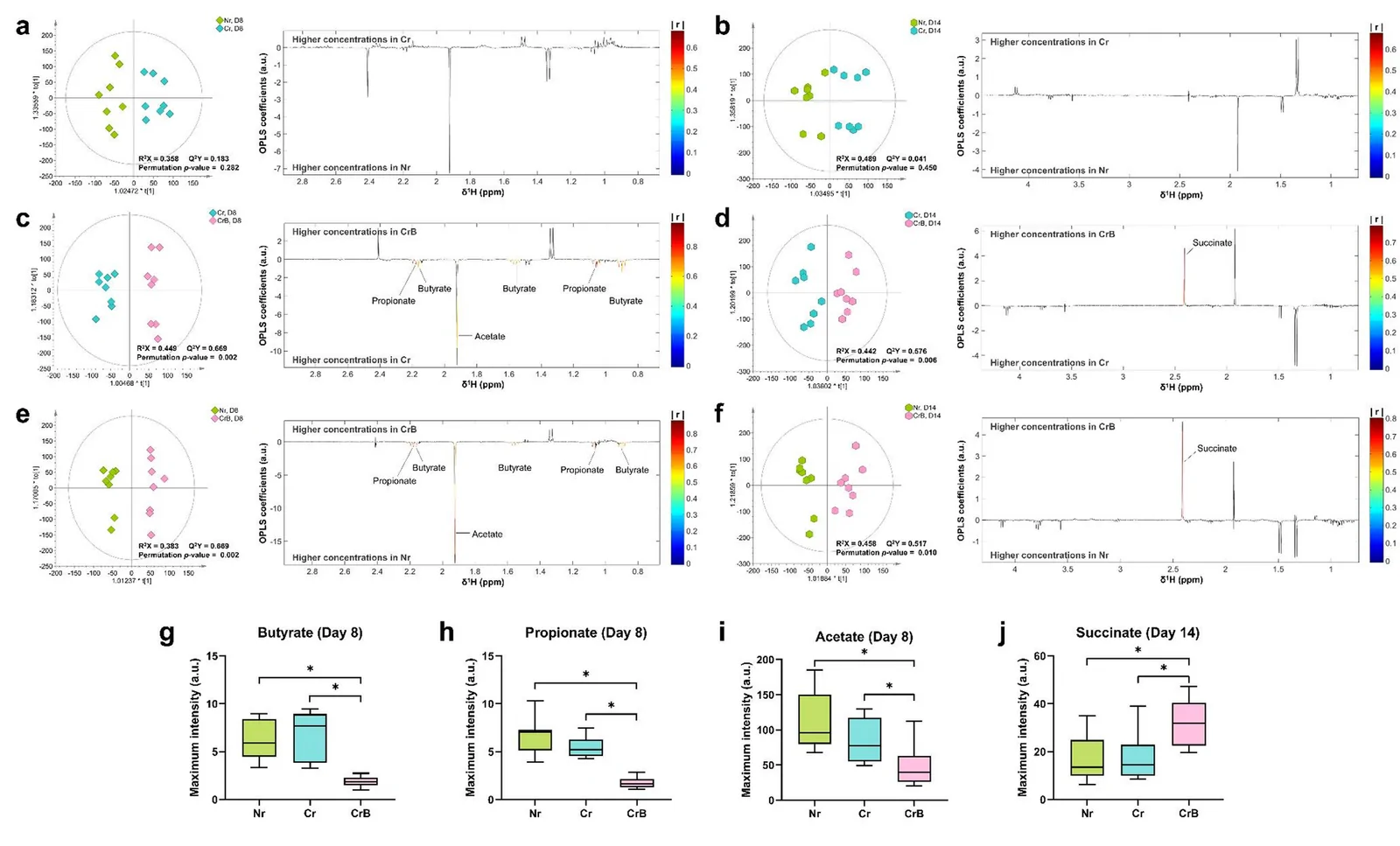
Orthogonal partial least squares discriminant analysis (OPLS-DA) scores plots and loadings plots of faecal metabolome data. Pairwise OPLS-DA scores plots and loadings plots of faecal metabolic profiles between Nr (green), Cr (blue), and CrB (pink) Wistar rats at (**a**,** c**,** e**) day 8 and (**b**,** d**,** f**) day 14. Peaks marked in red are metabolites significant after Benjamini-Hochberg correction (*p* < 0.05). Colour spectrum indicates OPLS-DA correlation coefficient |r|. Significant metabolites in faeces obtained from OPLS-DA models at day 8 (**g–i**) and day 14 (**j**). * indicates statistically significant between pairwise groups at p-value < 0.05 using Kruskal-Wallis test with Benjamini-Hochberg correction.

No significant differences in faecal metabolic profiles were observed between Nr and Cr groups at any time point (Table S2, permutation *p* > 0.05; Fig. 3a, b), indicating that CCA-associated microbiota did not produce readily detectable changes in intestinal metabolite output relative to healthy donor microbiota in this model. As the CrB group had not yet received bile at day 1, no differences between Cr and CrB were expected or observed at this time point (Table S2).

Significant faecal metabolomic differences were detected in CrB compared to Nr and Cr groups following bile reinfusion. At day 8, following the first bile gavage, Nr and Cr groups showed significantly higher faecal concentrations of SCFAs – butyrate, propionate, and acetate, compared to the CrB group (Fig. 3c, e, g, h, i; Kruskal-Wallis with BH correction, *p* < 0.05). As day-8 faecal samples were collected after bile administration, this rapid SCFA reduction is attributable to the acute effects of the initial bile dose on microbial fermentation activity, consistent with known bile acid-mediated suppression of SCFA-producing bacterial taxa. At day 14, CrB group showed significantly higher faecal succinate compared to both Nr and Cr groups (Fig. 3d, f, j; *p* < 0.05). Together, these data indicate that bile reinfusion was associated with a distinct shift in intestinal fermentation-associated metabolite profiles, characterised by initial reduction in SCFA output followed by succinate accumulation, which was not observed in the Nr or Cr groups at any time point.

### Plasma metabolic alterations following FMT and bile reinfusion

**Fig. 4:**
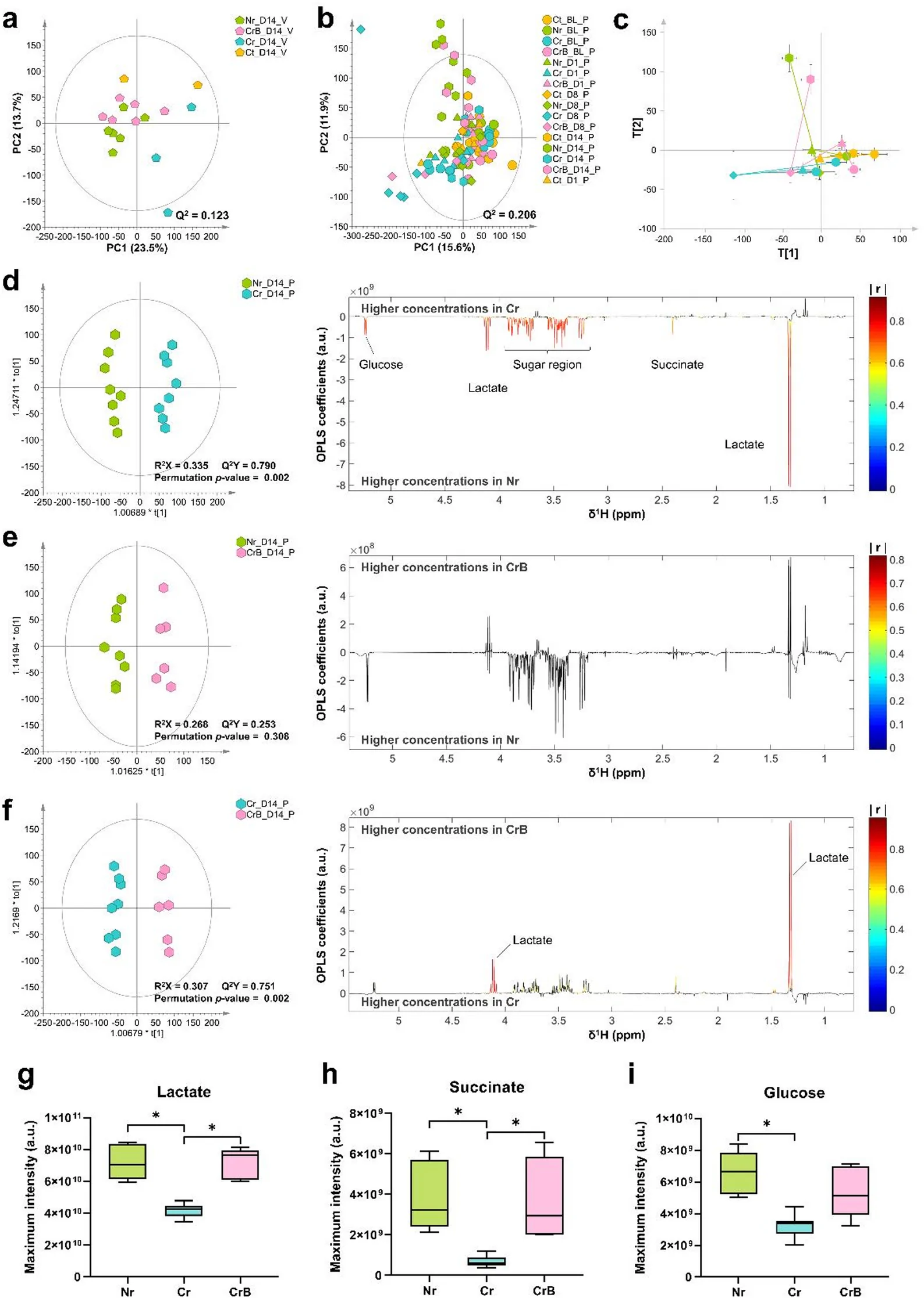
Principal component analysis of plasma metabolome data and pairwise OPLS-DA comparison models. (**a**) PCA scores plot of portal vein plasma at day 14. (**b**) PCA scores plot and (**c**) time-trajectory PCA scores plot of peripheral plasma metabolic profiles. (**d–f**) OPLS-DA scores plots and loadings plots of peripheral plasma metabolic profiles between Nr (green), Cr (blue), and CrB (pink) Wistar rats at day 14. (**g–i**) Significant metabolites in peripheral plasma obtained from OPLS-DA models at day 14. * indicates statistically significant between pairwise groups at p-value < 0.05 using Kruskal-Wallis test with Benjamini-Hochberg correction.

Plasma metabolites were identified by ¹H NMR spectroscopy (Fig. 1c; Table S1). PCA of plasma metabolic profiles confirmed clear separation of portal vein plasma from peripheral plasma (Fig. 2e). Portal vein plasma profiles of Nr and CrB groups were more similar to each other than to Cr at day 14 (Fig. 4a), whilst peripheral plasma showed the most pronounced group differences at day 14 (Fig. 4b-c). Pairwise OPLS-DA analyses with permutation validation were performed at day 1, day 8, and day 14 (Table S2), and significant metabolites were confirmed by Kruskal-Wallis with Benjamini-Hochberg (BH) correction.

No significant differences in peripheral plasma metabolic profiles were observed between any groups at day 1 or day 8 (Table S2; permutation *p* > 0.05). At day 14, the Cr group showed significant differences in peripheral plasma profiles compared to both Nr and CrB groups (Fig. 4d, f; *p* < 0.05), whilst Nr and CrB groups were not significantly different from each other (Fig. 4e). The peripheral plasma levels of glucose, lactate, and succinate were higher in Nr compared to Cr at day 14 (Fig. 4d, g, h, i; *p* < 0.05). CrB group also showed higher peripheral plasma lactate compared to Cr at day 14 (Fig. 4f, g; *p* < 0.05). The similarity of peripheral plasma metabolic profiles between CrB and Nr groups at day 14 is consistent with a bile reinfusion-associated convergence of systemic metabolic phenotypes; however, this should be interpreted with caution given the distinct faecal metabolomic profile of CrB described above. Portal vein plasma glucose, lactate, and succinate were higher in Nr than Cr at day 14, but these differences did not achieve statistical significance after BH correction (Fig. S3).

### Urinary metabolic alterations following FMT and bile reinfusion

Urinary metabolites were identified by ¹H NMR spectroscopy (Fig. 1b; Table S1). Pairwise OPLS-DA analyses revealed no statistically significant differences in urinary metabolic profiles between most group comparisons at any time point (Table S2; permutation *p* > 0.05). One exception was the Nr versus CrB comparison at day 14, which reached statistical significance (Fig. S4; permutation *p* < 0.05); however, no individual metabolites survived BH correction. These findings suggest that the metabolic effects of CCA-associated microbiota and bile reinfusion are primarily manifested in the intestinal and systemic circulating metabolic compartments rather than in renal excretion products.

### Faecal microbial community shifts following FMT and bile reinfusion

Full-length 16S rRNA gene Nanopore sequencing was used to characterise faecal microbial communities from Ct, Nr, Cr, and CrB groups at day − 6, 0, 1, and 14. Microbial community analyses were performed at OTU, family, and genus levels, including alpha diversity (Shannon diversity index) and beta diversity (Bray-Curtis dissimilarity with PERMANOVA), with pairwise comparisons between groups and across time points.

**Fig. 5:**
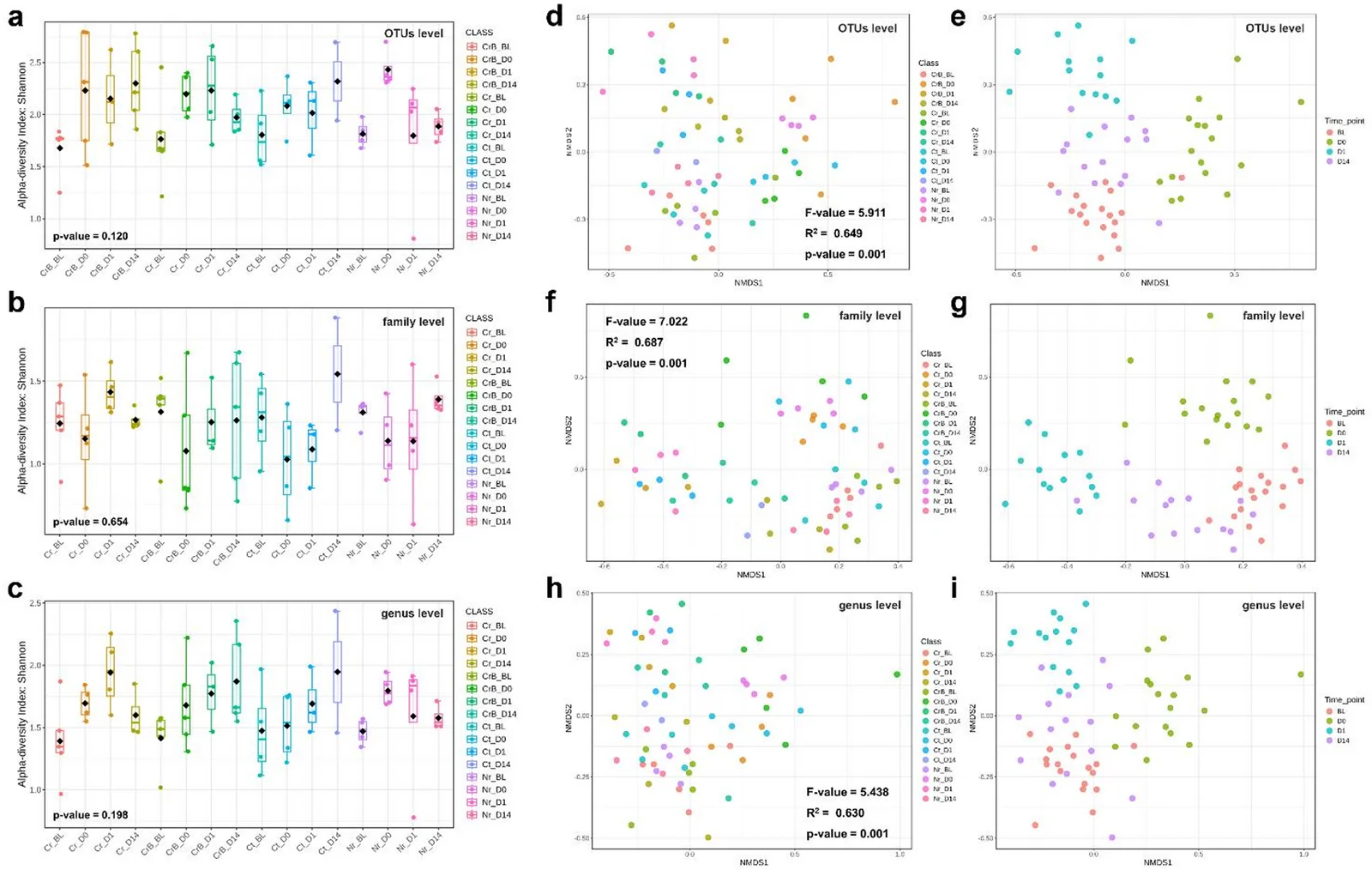
Microbial community analyses at OTUs, family and genus level. (**a–c**) Alpha diversity based on Shannon’s diversity. (**d–i**) Beta diversity based on Bray-Curtis dissimilarity and PERMANOVA. The same NMDS plots are shown with colors based on class (**d**,** f**,** h**) and time points (**e**,** g**,** i**).

Alpha diversity (Shannon index) did not differ significantly between groups at most time points (Fig. 5a-c, Fig. S5), suggesting that FMT from either donor type, and bile reinfusion, did not substantially alter overall species richness in the recipient rats. Beta diversity PERMANOVA indicated statistically significant differences in microbial community composition between groups across the study period (*p* < 0.05; Fig. 5d-i), consistent with successful engraftment and treatment-associated community shifts.

**Fig. 6:**
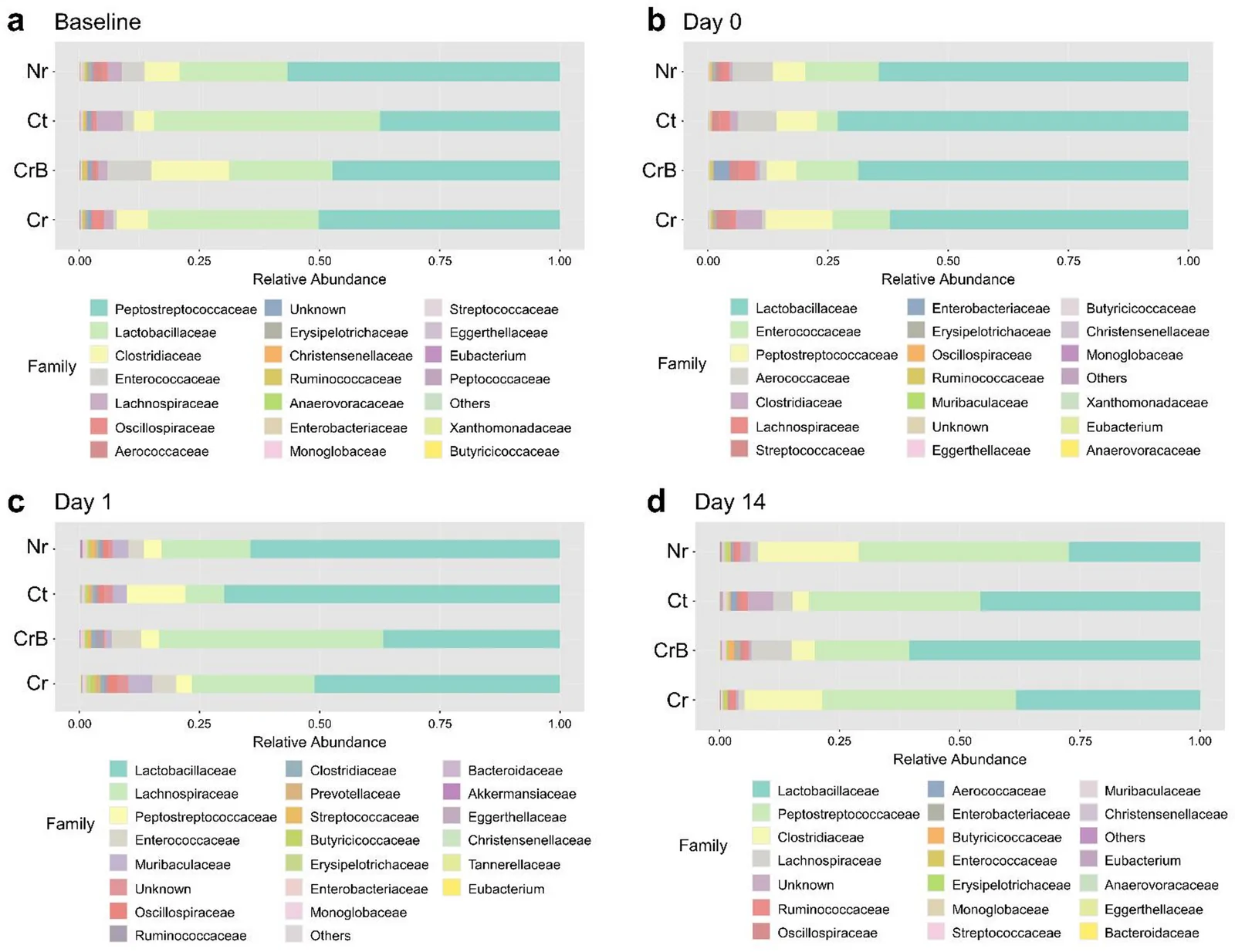
Relative abundance of faecal microbiota at family level. The relative abundance of faecal microbiota from Ct, Nr, Cr and CrB Wistar rats at (**a**) baseline, (**b**) day 0, (**c**) day 1, and (**d**) day 14.

**Fig. 7:**
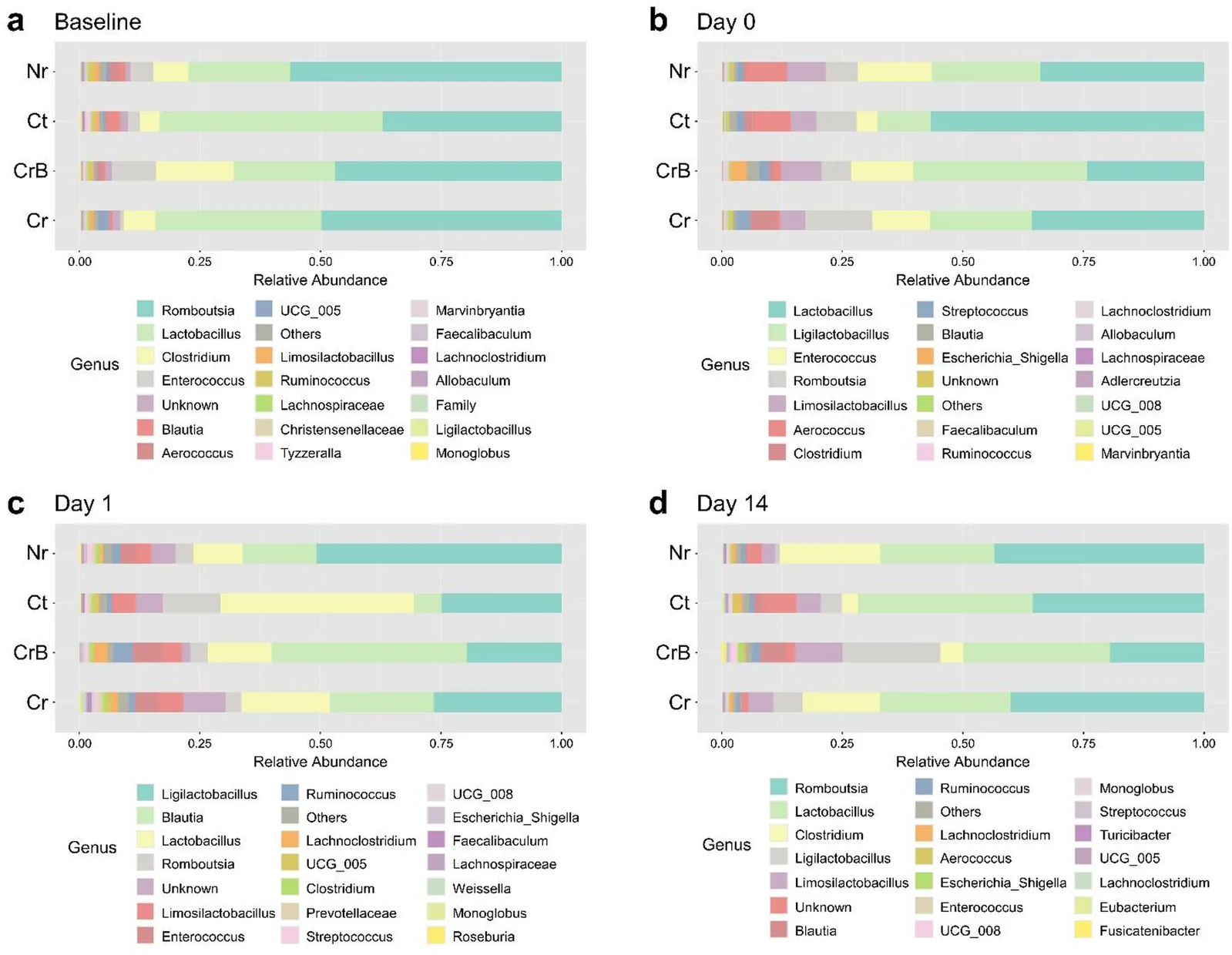
Relative abundance of faecal microbiota at genus level. The relative abundance of faecal microbiota from Ct, Nr, Cr and CrB Wistar rats at (**a**) baseline, (**b**) day 0, (**c**) day 1, and (**d**) day 14.

Relative abundance analysis at the family and genus levels demonstrated compositional changes over time (Figs. 6 and 7). At day 14, CrB group showed a substantially higher relative abundance of Lactobacillaceae (60.54%) compared to Nr (27.33%) and Cr (38.30%) groups (Fig. 6d; Table S3). Conversely, Nr and Cr groups showed higher relative abundance of Peptostreptococcaceae (43.76% and 40.35%, respectively) compared to CrB (19.68%). At the genus level, Romboutsia was most prevalent in Nr and Cr groups (43.52% and 40.10%, respectively), whereas Lactobacillus was most prevalent in CrB group (30.44%) (Fig. 7d; Table S4). These compositional differences, particularly the enrichment of Lactobacillaceae in CrB, are consistent with the altered fermentation metabolite profiles described above.

## Discussion

**Fig. 8:**
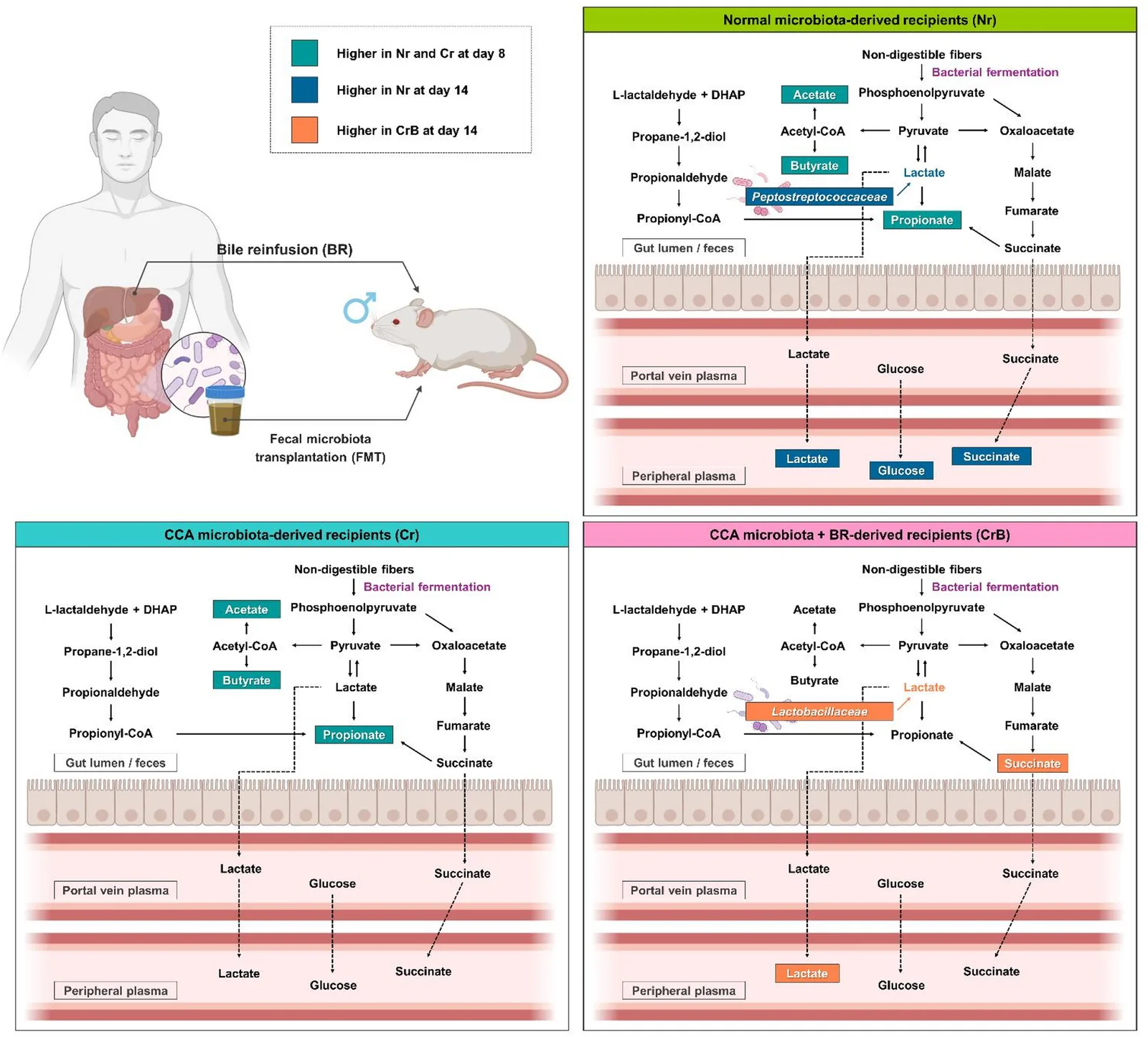
Schematic illustration of metabolic alterations observed in biofluids. Green box arrow indicates higher concentration of metabolites in Nr and Cr groups at day 8. Blue indicates higher concentration of metabolites in Nr group at day 14. Orange box indicates higher concentration of metabolites in CrB group at day 14.

Our findings characterise, for the first time, the metabolic phenotypes of faeces, plasma, and urine following CCA-derived faecal microbiota transplantation and bile reinfusion in a Wistar rat model. The results reveal both time-dependent and compartment-specific metabolic changes, with the most pronounced differences observed at day 14. Importantly, the pattern of findings – in which faecal and plasma metabolomic differences are dissociated between Nr/Cr and CrB groups – provides a nuanced picture of the relative contributions of microbiota composition and bile acid exposure to host metabolic phenotypes (Fig. 8).

A key observation requiring careful interpretation is the absence of significant faecal metabolomic differences between Nr and Cr groups at any time point, despite transplantation with microbiota from distinct donor populations (healthy donors vs. CCA patients). This finding suggests that, in the context of this rat FMT model, CCA-associated microbiota does not produce readily detectable changes in intestinal metabolite output relative to healthy donor microbiota. Several factors may underlie this observation. Antibiotic preconditioning, while necessary to deplete host microbiota prior to FMT, may have created an ecological niche that favoured convergent re-colonisation patterns regardless of donor origin. Furthermore, the engraftment of human microbiota into rat recipients is inherently incomplete owing to species-specific physiological and immunological differences that may buffer inter-donor functional diversity. Consistent with this, alpha diversity did not differ significantly between Nr and Cr groups, suggesting that both donor microbiota reached broadly similar species richness in the recipient hosts. These are important limitations of rodent FMT models that we acknowledge explicitly.

Despite the absence of faecal metabolomic differences, the Cr group showed significantly lower peripheral plasma levels of glucose, lactate, and succinate compared to Nr at day 14. This dissociation between faecal and plasma metabolomics is biologically meaningful: host systemic metabolic regulation is determined not only by luminal microbial metabolite production, but also by microbial-host interactions at mucosal, hepatic portal, and systemic levels that may diverge from simple faecal metabolite output. These findings are consistent with the possibility that CCA-associated microbiota may influence host systemic metabolism through pathways not readily captured by faecal metabolomics in this model. However, causal inferences cannot be drawn from these associative data, and mechanistic validation through germ-free models or targeted functional assays would be required.

In contrast to the Nr-Cr comparison, bile reinfusion was associated with marked changes in both the faecal and microbiome compartments. Faecal levels of SCFAs including butyrate, propionate, and acetate were lower in CrB compared to Nr and Cr groups at day 8, suggesting that early bile reinfusion is associated with reduced intestinal SCFA production. SCFAs, the primary products of anaerobic microbial fermentation of dietary polysaccharides, play crucial roles in maintaining intestinal homeostasis and host energy metabolism^16,17^. The suppression of SCFA output in CrB at day 8 is consistent with bile acid-mediated shifts in microbial community composition and fermentation capacity at this early time point.

Following continued daily bile reinfusion, faecal succinate was significantly elevated in CrB compared to Nr and Cr groups at day 14. Both SCFA production and succinate accumulation reflect the activity of gut microbial fibre fermentation pathways, though at different stages^18,19^. The progression from reduced SCFAs at day 8 to elevated succinate at day 14 in CrB is consistent with bile-driven dysregulation of microbial fermentation, potentially reflecting an imbalance between succinate-producing and succinate-consuming bacteria resulting from the bile acid-associated enrichment of Lactobacillaceae at the expense of Peptostreptococcaceae^20–22^. Succinate accumulation in the gut lumen has been associated with intestinal inflammation and microbiota perturbation in conditions such as inflammatory bowel disease^18–20^, although whether similar inflammatory consequences occur in the context of bile reinfusion in CCA remains to be investigated. Notably, these faecal metabolic changes in CrB were distinct from the Nr group, arguing against a simple “restoration” of healthy intestinal fermentation by bile reinfusion. Rather, bile reinfusion appears to drive a distinct remodelling of the intestinal metabolic environment.

Despite distinct faecal metabolic profiles, peripheral plasma metabolic phenotypes of CrB and Nr groups were not significantly different at day 14, whilst the Cr group diverged significantly from both. This apparent paradox – faecal difference without plasma difference between CrB and Nr, but plasma difference without faecal difference between Cr and Nr – highlights an important principle: the faecal and plasma metabolomes capture complementary but partially distinct aspects of host-microbiota co-metabolism. The faecal metabolome reflects intestinal microbial metabolic activity, whilst the plasma metabolome integrates intestinal, hepatic, and peripheral contributions. Bile acids are known to act as signalling molecules at multiple sites along the gut-liver axis, activating nuclear receptors and G-protein-coupled receptors (including TGR5) that regulate hepatic and peripheral metabolic processes independently of faecal metabolite profiles^10,11^. We propose that the convergence of CrB and Nr plasma profiles may reflect bile acid-mediated systemic signalling that overrides the divergent intestinal metabolic environments of these two groups. This remains a speculative, hypothesis-generating interpretation: future studies incorporating bile acid profiling, bile acid receptor signalling assays, and germ-free models would be required to test this mechanistic framework.

The connection between peripheral plasma lactate levels and the microbial community composition in the CrB group warrants specific attention. Lactate plays important roles in host-microbe metabolic interactions and in energy metabolism^23,24^. The enrichment of Lactobacillaceae – a diverse family of lactic acid-producing bacteria possessing the positive impacts on gut health, including regulating the immune system and offering defence against infections^25^– in CrB group at day 14, associated with higher peripheral plasma lactate compared to Cr group, is consistent with a microbiota-to-host lactate axis, though the quantitative contribution of gut microbial lactate production to circulating levels requires experimental validation. The lower peripheral plasma glucose observed as a numerical trend in CrB compared to Nr – a difference that did not reach statistical significance – is broadly consistent with published observations that bile acids can modulate glucose metabolism and appetite suppression through TGR5-mediated signalling^26–28^; however, this non-significant numerical trend should not be over-interpreted. Food intake and hormonal parameters (e.g., GLP-1, GIP) were not measured in the current study, and whilst body weight did not differ significantly between groups (Fig. S1), the mechanistic contribution of TGR5-mediated pathways to the observed trend in plasma glucose cannot be substantiated from our data. This interpretation remains purely speculative and hypothesis-generating, warranting dedicated investigation in future studies with appropriately powered designs.

Regarding the full-length 16S rRNA Nanopore sequencing approach: a key advantage of this method is the superior taxonomic resolution conferred by V1–V9 amplification, enabling genus-level and, in many cases, species-level classification compared to short-read amplicon approaches. The use of the super-accuracy (SUP) Dorado basecalling model minimised per-read error rates. A recognised limitation of Oxford Nanopore sequencing is the higher raw error rate compared to short-read platforms, and all taxonomic assignments should be considered at the resolution supported by the reference database (SILVA 138.1). Additionally, as with all amplicon-based approaches, our data reflect relative rather than absolute microbial abundance, precluding conclusions about total microbial biomass. Future studies using absolute quantification approaches (e.g., spike-in controls) would complement the current relative abundance data.

In summary, our findings indicate that bile reinfusion in the context of CCA-associated faecal microbiota transplantation is associated with shifts in gut microbial composition (enrichment of Lactobacillaceae), altered intestinal fermentation metabolite profiles (decreased SCFAs and increased succinate), and a convergence of peripheral plasma metabolic phenotypes towards those observed in healthy microbiota recipients. Notably, CCA-associated microbiota alone was associated with systemic but not intestinal metabolic differences compared to healthy microbiota, pointing to a potentially greater contribution of bile acid exposure than microbiota composition per se to intestinal metabolic outcomes in this model. These associations highlight the potential importance of the bile acid–microbiota axis in modulating host-microbiota metabolic interactions in CCA and provide a basis for mechanistic investigation and the development of microbiota- and bile-targeted therapeutic strategies.

## Conclusion

Our findings demonstrate that CCA-associated gut microbiota is associated with altered systemic metabolic profiles compared to healthy microbiota in a rat FMT model, as evidenced by significant differences in peripheral plasma metabolites. Notably, no significant faecal metabolomic differences were observed between CCA and healthy microbiota recipient groups, suggesting that CCA microbiota-associated systemic metabolic differences may not be directly reflected in intestinal metabolite output in this experimental system, and pointing to bile acid exposure as a potentially dominant modulator of intestinal metabolic phenotypes. Bile reinfusion was associated with shifts in gut microbial composition, with enrichment of Lactobacillaceae, altered intestinal fermentation metabolites, and a convergence of peripheral plasma profiles towards those of healthy microbiota recipients. These findings highlight the bile acid-microbiota axis as a target for further mechanistic investigation and for developing monitoring and treatment strategies in CCA and related hepatobiliary disorders.

## Methods

### Animal study and sample collection

All animal experiments were approved by the Institutional Animal Care and Use Committee of Khon Kaen University (IACUC-KKU-48/65) and conducted in accordance with IACUC-KKU and ARRIVE guidelines. Thirty-two male Wistar rats aged 6 weeks (mean body weight 186 ± 23 g) were obtained from the Northeast Laboratory Animal Center (NELAC), Khon Kaen University, Thailand, and acclimatised for one week. Animals were randomly housed in groups of four per cage under the controlled conditions (22 °C in a 12 h light/dark cycle) and divided into four groups: (1) control (Ct, *n* = 8): received sterile 1X PBS without faecal microbiota; (2) normal (Nr, *n* = 8): received faecal microbiota from participants with normal bile duct; (3) CCA (Cr, *n* = 8): received CCA faecal microbiota; and (4) CCA with bile (CrB, *n* = 8): received CCA faecal microbiota followed by daily bile reinfusion (Fig. S6).

All rats received 2 mg/100 g body weight antibiotic cocktail (ampicillin, metronidazole and neomycin, by oral gavage) one day prior to FMT to deplete intestinal microbiota (Fig. S6). Faecal samples from participants with normal bile duct and CCA patients, and bile from CCA patients, were obtained from a previous study by Treeriya et al., (2023), with Khon Kaen University Ethics Committee (KKUEC) approval (EC number: HE571283); all methods were conducted in accordance with the KKUEC guidelines, and informed consent was obtained from all participants. Faecal samples were pooled across donors, dissolved in sterile 1X PBS, filtered, and subsequently administered to Wistar rats by oral gavage.

After FMT, rats in the CrB group were administered bile, drained and pooled from multiple CCA patients, by oral gavage once daily from day 8 to day 14. On each sampling day (day 8 and day 14), bile was administered in the morning prior to faecal and biofluid sample collection, such that collected samples reflect the post-dose intestinal environment (Fig. S6). Rats were anaesthetised by intraperitoneal injection with thiopental sodium (80 mg/kg) and cardiac blood was collected as a terminal procedure. Faecal, urinary and peripheral blood samples were collected at day − 6 (baseline), 0, 1, 8 and 14. Portal vein blood samples were collected at the sacrifice time point.

### ^1^H NMR spectroscopic analysis

Approximately 200 mg of faeces were combined with 565 µL of HPLC-grade water, vortexed for 15 min and centrifuged at 10,000 x g for 5 min. A total of 400 µL faecal water extract were mixed with 640 µL of 0.075 M sodium phosphate buffer (0.08% TSP, 4% NaN_3_, pH 7.4), vortexed, centrifuged at 12,000 x g for 10 min at 4 °C and 550 µL of supernatant was transferred into a 5 mm NMR tube.

Urine samples were centrifuged at 12,000 g for 10 min at 4 °C, and 540 µL of supernatant was mixed with 60 µL of 0.075 M sodium phosphate buffer (0.08% TSP, 4% NaN_3_, pH 7.4). The mixture was vortexed, centrifuged at 12,000 g for 10 s at 4 °C, and 580 µL transferred to a 5 mm NMR tube.

Plasma samples were centrifuged at 12,000 g for 10 min at 4 °C, and 300 µL of plasma was mixed with 300 µL of 0.075 M sodium phosphate buffer (0.002% TSP, 4% NaN_3_, pH 7.4), vortexed, centrifuged at 12,000 g for 10 s at 4 °C, and 580 µL transferred to a 5 mm NMR tube.

^1^H NMR spectra were acquired on a 400 MHz NMR spectrometer with CryoProbe (Bruker, USA) using the Carr−Purcell−Meiboom−Gill (CPMG) pulse sequence [RD − 90˚−(τ − 180˚−τ)n−acquisition] at 310 K with 64 scans.

### ^1^H NMR spectral data pre-processing and metabolite identification

Phasing, baseline correction, and chemical shift referencing to TSP (δ^1^H 0) in the faecal and urinary spectra, and to the anomeric proton of α-glucose (δ^1^H 5.223) in the plasma spectra, were automatically performed in TopSpin software (Bruker, USA). Data were imported to MATLAB (MathWorks, USA) for pre-processing. Water peak and noise regions were excised: urea signals were removed from urinary spectra. All spectra were aligned using the recursive segment-wise peak alignment (RSPA) method. Faecal and urinary spectra were normalised using probabilistic quotient normalization (PQN); plasma spectral were not normalised. Statistical total correlation spectroscopy (STOCSY) was used to assist metabolite assignment^29^. Resonances were matched against Chenomx software (Chenomx Inc., Edmonton, Canada) and the Human Metabolome Database (HMDB)^30–34^.

### ^1^H NMR spectral data statistical analysis

Pre-processed spectral data were imported into SIMCA version 14.1 (Umetrics Inc., Sweden) for PCA and OPLS-DA with a unit variance (UV) scaling. OPLS-DA scores and coefficient loadings plots were constructed in MATLAB R2015a environment (MathWorks, USA). Models used mean-centred and UV-scaled data with one orthogonal component and one PLS component. Loadings were colour-coded by correlation coefficient values |r|. Model validity was assessed by R^2^, Q^2^ and permutation p-values (999 permutations; *p* < 0.05). To complement multivariate findings, individual metabolites identified as discriminatory by OPLS-DA were further evaluated by Kruskal-Wallis test with Benjamini-Hochberg false discovery rate correction (*p* < 0.05). PCA-based PERMANOVA was performed to assess overall metabolic profile separation between groups. OPLS-DA is used as an exploratory tool for biomarker identification; statistical significance of individual metabolites is determined by the accompanying univariate analyses.

### Full-length 16S rRNA gene sequencing analysis

Bacterial genomic DNA from faeces was extracted using the ZymoBIOMICS™ DNA Miniprep Kit (D4300, Zymo Research Corp., Irvine, CA, United States). Approximately 250 mg of faeces was processed in ZR BashingBead™ Lysis Tubes with 750 µL ZymoBIOMICS™ Lysis Solution, bead-beaten at 2,000 rpm for 5 min, and centrifuged at 10,000 x g for 1 min. DNA concentration was measured using a Qubit 4.0 fluorometer (Thermo Fisher Scientific, Waltham, Massachusetts, US).

Full-length (V1-V9) 16S rRNA gene amplification was performed using the 16S Barcoding Kit (SQK-16S024, Oxford Nanopore Technologies, UK), which includes 27 F/1492R primers. Libraries were prepared per manufacturer instructions, pooled at equal concentrations, ligated with rapid adaptors and sequenced on a FLO-MIN106 R9.4.1 flow cell using the MinION sequencer (MinKNOW platform). This approach confers superior taxonomic resolution at genus and species level compared to short-read amplicon sequencing, owing to full-length amplification; recognised limitations include higher per-read error rates compared to short-read platforms, which were mitigated by super-accuracy (SUP) basecalling in Dorado (Oxford Nanopore Technologies), and the generation of relative rather than absolute abundance data.

Raw POD5 files were base-called using Dorado basecaller (Oxford Nanopore Technologies, UK) with super-accuracy (SUP). Filtered reads were taxonomically assigned using Minimap 2^35,36^ against SILVA database (version 138.1)^37–39^. Data exploration, diversity analyses, and visualisation were performed using MicrobiomeAnalyst 2.0^40^. Alpha diversity (Shannon diversity index) was calculated and compared between groups by Kruskal-Wallis test. Beta diversity based on Bray-Curtis dissimilarity was assessed by NMDS ordination with PERMANOVA for significance testing.

## Supplementary Information

Below is the link to the electronic supplementary material.

Supplementary Material 1

## Data Availability

The datasets generated during the current study are available in the Open Science Framework (OSF) repository, https://osf.io/ewbh7.
