# Supplementary material for: Bile reinfusion is associated with shifts in host-microbiota metabolic profiles following cholangiocarcinoma-associated microbiota transplantation
Source: Sci Rep. 2026 Jun 26;16:29432. doi: 10.1038/s41598-026-58698-2 (PMC13594166; doi:10.1038/s41598-026-58698-2)
Supplement: Supplementary file 1 [file 41598_2026_58698_MOESM1_ESM.docx]

**Supporting information for**

**Bile Reinfusion Is Associated with Shifts in Host-Microbiota Metabolic Profiles Following Cholangiocarcinoma-Associated Microbiota Transplantation**

**Thanaporn Saensupha**^1^**, Anyarin Jirahiranpat**^1^**, Natthawut Pratumwan**^1,2^**, Theerayut Bubpamala**^1,2^**, Wassana Jamnongkan**^2^**, Thanaporn Kulthawatsiri**^3^**, Manida Suksawat**^2,4^**, Kanokrat Thaiwatcharamas**^5^**, Waraporn Auiewiriyanukul**^6^**, Marisa Attapong**^2^**, Navaporn Sritong**^2^**, Benjamin H Mullish**^7,8,*^**, and Jutarop Phetcharaburanin**^2,3,*^

1 Medical Biochemistry and Molecular Biology Graduate Study Program, Faculty of Medicine, Khon Kaen University, Khon Kaen, Thailand

2 Khon Kaen University National Phenome Institute, Office the President, Khon Kaen University, Khon Kaen, Thailand

3 Department of Systems Biosciences and Computational Medicine, Faculty of Medicine, Khon Kaen University, Khon Kaen, Thailand

^4^ International College, Khon Kaen University, Khon Kaen, Thailand

5 Department of Surgery, Faculty of Medicine, Khon Kaen University, Khon Kaen, Thailand

6 Department of Biochemistry, Faculty of Science, Kasetsart University, Bangkok, Thailand

^7^ Division of Digestive Diseases, Department of Metabolism, Digestion and Reproduction, Faculty of Medicine, Imperial College London, London, UK

^8^ Department of Hepatology, St Mary’s Hospital, Imperial College Healthcare NHS Trust, London, UK

^*^Corresponding authors: [jutarop@kku.ac.th](mailto:jutarop@kku.ac.th); b.mullish@imperial.ac.uk

**Figure S1** Body weight of control (Ct), normal (Nr), CCA (Cr), and CCA with bile (CrB) groups of Wistar rats at baseline, day 0, day 1, day 8, and day 14. The data are shown as mean ± SD and statistical differences determined by two-way ANOVA. * indicates statistically significant between pairwise groups at p-value < 0.05.

**Figure S2** PCA-based PERMANOVA of metabolic profiles from feces (**a**), plasma (**b**), and urine (**c**).

**Figure S3** Orthogonal partial least squares discriminant analysis (OPLS-DA) scores plots (left panel) and loadings plots (right panel) of portal vein plasma metabolic profiles between (**a**) Nr (green) vs. Cr (blue), (**b**) Nr (green) vs. CrB (pink), and (**c**) Cr (blue) vs. CrB (pink) Wistar rats at day 14. The color spectrum indicates OPLS-DA correlation coefficients |r|.

**Figure S4** Orthogonal partial least squares discriminant analysis (OPLS-DA) scores plots (left panel) and loadings plots (right panel) of urinary metabolic profiles between (**a**) Nr (green) vs. Cr (blue), (**b**) Nr (green) vs. CrB (pink), and (**c**) Cr (blue) vs. CrB (pink) Wistar rats at day 14. The color spectrum indicates OPLS-DA correlation coefficients |r|.

**Figure S5** Alpha diversity based on Shannon’s diversity at family level at baseline (**a**), day 0 (**b**), day 1 (**c**), and day 14 (**d**). Pairwise comparisons of alpha diversity were conducted using Kruskal-Wallis test.

**Figure S6**	Timeline for animal experiments and study design.

**Table S1**	List of identified metabolites

| **No.** | **Metabolite** | **Chemical shift** | **Biofluid** |
| --- | --- | --- | --- |
| 1 | Butyrate | 0.899(t); 1.554(q); 2.162(t) | F |
| 2 | Isoleucine | 0.942(t); 1.003(d); 1.243(m); 1.987(m); 3.672(d) | F, P |
| 3 | Isovalerylglycine | 0.934(d); 1.98(m); 2.167(d); 3.74(d) | U |
| 4 | Leucine | 0.951(m); 0.966(m); 1.731(m); 3.737(t) | F, P |
| 5 | Valine | 0.985(d); 1.038(d); 2.273(m); 3.611(d) | F, P |
| 6 | Propionate | 1.060(t); 2.178(q) | F |
| 7 | 3-Methyl-2-oxovalerate | 1.094(d); 2.928(m); 1.461(m); 0.899(t) | F |
| 8 | Propylene glycol | 1.137(d); 3.460(dd); 3.542(dd) | F |
| 9 | Ethanol | 1.179(t); 3.646(q) | P |
| 10 | 3-Hydroxybutyrate | 1.187(d); 2.310(dd); 2.377(dd); 4.159(td) | P |
| 11 | Methylmalonate | 1.243(d); 3.16(q) | F |
| 12 | Lactate | 1.326(d); 4.115(q) | F, P |
| 13 | Threonine | 1.326(d); 3.589(d); 4.260(m) | F |
| 14 | Dimethylmalonate | 1.442(s) | F, U |
| 15 | Lysine | 1.493(m); 1.731(m); 1.90(dtd); 2.12(dtd); 3.031(t); 3.762(t) | F, P |
| 16 | Alanine | 1.475(d); 3.780(m) | F, P |
| 17 | 5-Aminopentanoate | 1.650(m); 2.238(t); 3.021(t) | F |
| 18 | 4-Aminobutyrate | 1.904(m); 2.302(t); 3.021(t) | F |
| 19 | Acetate | 1.923(s) | F, U, P |
| 20 | Acetone | 2.227(s) | P |
| 21 | Glutamate | 2.048(m); 2.115(dt); 2.358(dt); 3.762(t) | F |
| 22 | Pyruvate | 2.368(s) | P |
| 23 | Oxalacetate | 2.382(s) | U |
| 24 | Succinate | 2.411(s) | F, U, P |
| 25 | Glutamine | 2.444(dt); 2.452(dt); 2.120(td); 3.76(t) | P |
| 26 | Oxoglutarate | 2.449(s); 3.015(t) | U |
| 27 | Citrate | 2.553(d); 2.651(d) | U, P |
| 28 | Methylamine | 2.607(s) | F, U |
| 29 | Methionine | 2.127(m); 2.141(s); 2.648(t); 3.848(dd) | F |
| 30 | Aspartate | 2.692(dd); 2.795(dd); 3.891(dd) | F |
| 31 | Dimethylamine | 2.726(s) | F, U |
| 32 | Trimethylamine | 2.893(s) | F, P |
| 33 | Tyramine | 2.928(t); 3.232(t); 6.891(ddd); 7.208(ddd) | F |
| 34 | Creatine | 3.032(s); 3.927(s) | U, P |
| 35 | 1,1-dimethylbiguanide | 3.042(s) | U |
| 36 | Malonate | 3.136(s) | F |
| 37 | Dimethyl sulfone | 3.147(s) | U, P |
| 38 | Choline | 3.204(s) | F, P |
| 39 | Glycerophosphocholine | 3.233(s) | F |
| 40 | Taurine | 3.432(t); 3.266(t) | F, U |
| 41 | Trimethylamine N-oxide | 3.271(s) | U |
| 42 | Proline | 3.354(ddd); 2.014(m) | F |
| 43 | Methanol | 3.363(s) | F, P |
| 44 | Glycine | 3.566(s) | F, U, P |
| 45 | Dimethylglycine | 3.724(s); 2.920(s) | F, U |
| 46 | Guanidoacetate | 3.801(s) | U |
| 47 | Glycyl-glycine | 3.834(s); 3.845(s) | U |
| 48 | Betaine | 3.896(s); 3.251(s) | P |
| 49 | Creatinine | 4.053(s); 3.03(s) | U |
| 50 | Glycolate | 3.952(s) | F |
| 51 | Serine | 3.979(dd); 3.959(dd); 3.849(dd) | F |
| 52 | Trigonelline | 4.438(s); 8.835(m); 9.122(s) | U |
| 53 | Arabinose | 5.301(d); 5.240(d); 4.512(d); 4.00(ddd); 3.87(dd); 3.81(dd); 3.68(dd) | F |
| 54 | Xylose | 5.198(d); 4.571(d); 4.00(ddd); 3.85(dd); 3.68(dd); 3.52(dd) | F |
| 55 | Glucose | 5.228(d); 3.881(dd); 3.830(ddd); 3.856(m); 3.772(m); 3.732(dd); 3.715(t); 3.541(dd); 3.490(t); 3.456(ddd); 3.412(t); 3.400(t); 3.241(dd) | F, P |
| 56 | Erythrose | 5.268(d); 4.582(d); 4.12(dt); 3.87(dd); 3.52(dd) | F |
| 57 | Allantoin | 5.397(s) | U |
| 58 | Maleate | 6.014(s) | P |
| 59 | Orotate | 6.194(s) | F, U |
| 60 | Fumarate | 6.525(s) | F, U, P |
| 61 | Desaminotyrosine | 6.742(ddd); 2.844(t); 2.473(t) | F |
| 62 | Quinone | 6.800(s) | F |
| 63 | Tyrosine | 7.186(d); 6.891(d); 3.181(dd); 3.062(dd) | F, P |
| 64 | Phenylacetylglycine | 7.408(m); 7.366(m); 3.755(d) | U |
| 65 | Indoxyl sulfate | 7.694(ddd); 7.494(ddd); 7.361(s) | U |
| 66 | Phenylalanine | 7.416(m); 7.379(m); 7.324(m); 3.98(t); 3.27(dd); 2.84(dd) | F, P |
| 67 | Uracil | 7.530(d); 5.797(d) | F |
| 68 | Tryptophan | 7.725(d); 7.323(d); 7.286(ddd); 7.208(ddd); 4.04(t); 3.47(dd); 3.29(dd) | F, P |
| 69 | Hippurate | 7.829(dd); 7.642(tt); 7.552(m); 3.966(d) | U |
| 70 | Xanthine | 7.939(s) | F, U, P |
| 71 | Hypoxanthine | 8.216(s); 8.192(s) | F |
| 72 | Formate | 8.460(s) | F, U, P |
| 73 | Nicotinate | 8.942(dd); 8.606(dd); 8.248(ddd) | F |
| 74 | 1-Methylnicotinamide | 9.268(s); 8.949(d); 8.880(d); 8.177(t); 4.475(s) | U |
| 75 | Tartrate | 4.336(s) | U |
| 76 | Lipid (CH_3_−(CH_2_)_n_− of LDL&VLDL) | 0.863(bs) | P |
| 77 | Lipid (CH_3_−(CH_2_)_n_− of LDL&VLDL) | 1.273(bs) | P |
| 78 | Lipid (−CH_2_−CH_2_−C=O) | 1.566(bs) | P |
| 79 | Lipid (−CH_2_−CH=CH−) | 2.016(bs) | P |
| 80 | Lipid (−CH_2_−C=O) | 2.234(bs) | P |
| 81 | Lipid (=CH−CH_2_−CH=) | 2.747(bs) | P |
| 82 | Lipid (−CH=CH−) | 5.296(bs) | P |

**Abbreviations:** F: Feces; U: Urine; P: Peripheral and Portal vein plasma; s: singlet; d: doublet; dd: doublet of doublets; ddd: doublet of doublets of doublets; dt: doublet of triplet; dtd: doublet of triplet of doublets; t: triplet; td; triplet of doublets, q: quartet; m: multiplet; bs: broad singlet

**Table S2**	A summary of the PCA and OPLS-DA statistical parameters for pairwise comparisons of metabolome datasets. Significant models are bold.

| **Biofluid** | **Models** | **Time-point** | **PCA** | | | **OPLS-DA** | | | |
| --- | --- | --- | --- | --- | --- | --- | --- | --- | --- |
|  |  |  | **R^2^** | | **Q^2^** | **R^2^X** | **R^2^Y** | **Q^2^Y** | **Permutation *p*-value** |
|  |  |  | **PC1** | **PC2** |  |  |  |  |  |
| **Feces** | Nr *vs.* Cr | Day 1 | 29.9% | 15.6% | 0.205 | 37.8% | 81.6% | 0.014 | 0.466 |
|  | Nr *vs.* Cr | Day 8 | 28.3% | 14.8% | 0.135 | 35.8% | 87.5% | 0.183 | 0.282 |
|  | Nr *vs.* Cr | Day 14 | 41.3% | 9.77% | 0.299 | 48.9% | 81.9% | 0.041 | 0.450 |
|  | Nr *vs.* CrB | Day 1 | 22.7% | 16.0% | -0.018 | 27.7% | 89.7% | -0.138 | 0.562 |
|  | **Nr *vs.* CrB** | **Day 8** | **29.5%** | **14.8%** | **0.223** | **38.3%** | **96.2%** | **0.699** | **0.002** |
|  | **Nr *vs.* CrB** | **Day 14** | **36.3%** | **11.6%** | **0.296** | **45.8%** | **91.2%** | **0.517** | **0.010** |
|  | Cr *vs.* CrB | Day 1 | 26.8% | 11.9% | 0.075 | 33.5% | 92.3% | 0.016 | 0.362 |
|  | **Cr *vs.* CrB** | **Day 8** | **34.7%** | **10.5%** | **0.226** | **44.9%** | **95.5%** | **0.669** | **0.002** |
|  | **Cr *vs.* CrB** | **Day 14** | **34.6%** | **13.4%** | **0.275** | **44.2%** | **89.3%** | **0.576** | **0.006** |
| **Urine** | Nr *vs.* Cr | Day 1 | 15.3% | 12.0% | -0.023 | 15.8% | 98.4% | -9.94x10^-4^ | 0.824 |
|  | Nr *vs.* Cr | Day 8 | 17.8% | 15.9% | -0.059 | 23.8% | 93.6% | 0.243 | 0.172 |
|  | Nr *vs.* Cr | Day 14 | 19.4% | 12.1% | 0.034 | 27.1% | 94.5% | 0.421 | 0.300 |
|  | Nr *vs.* CrB | Day 1 | 15.4% | 12.8% | 0.002 | 21.6% | 97.5% | 0.260 | 0.138 |
|  | Nr *vs.* CrB | Day 8 | 17.8% | 12.9% | 0.013 | 22.5% | 92.8% | 0.341 | 0.236 |
|  | **Nr *vs.* CrB** | **Day 14** | **23.2%** | **13.2%** | **0.038** | **22.0%** | **95.3%** | **0.536** | **0.040** |
|  | Cr *vs.* CrB | Day 1 | 14.9% | 13.6% | -0.005 | 21.7% | 96.5% | 0.322 | 0.248 |
|  | Cr *vs.* CrB | Day 8 | 17.9% | 13.3% | -0.098 | 20.8% | 95.2% | 0.154 | 0.304 |
|  | Cr *vs.* CrB | Day 14 | 23.4% | 12.0% | 0.052 | 32.3% | 83.1% | 0.254 | 0.602 |
| **Plasma** | Nr (P) *vs.* Cr (P) | Day 1 | 22.7% | 13.6% | 0.035 | 28.9% | 97.8% | 0.405 | 0.412 |
|  | Nr (P) *vs.* Cr (P) | Day 8 | 26.2% | 16.3% | 0.084 | 34.5% | 91.5% | 0.328 | 0.368 |
|  | **Nr (P) *vs.* Cr (P)** | **Day 14** | **23.4%** | **16.7%** | **0.191** | **33.5%** | **96.8%** | **0.790** | **0.002** |
| **Plasma** | Nr (P) *vs.* CrB (P) | Day 1 | 21.5% | 12.8% | 0.032 | 28.6% | 95.6% | 0.257 | 0.582 |
|  | Nr (P) *vs.* CrB (P) | Day 8 | 23.2% | 13.2% | 0.072 | 30.5% | 96.2% | 0.354 | 0.172 |
|  | Nr (P) *vs.* CrB (P) | Day 14 | 19.1% | 15.4% | 0.052 | 26.8% | 95.3% | 0.253 | 0.308 |
|  | Nr (V) *vs.* CrB (V) | Day 14 | 20.0% | 16.3% | 0.022 | 30.5% | 98.0% | 0.668 | 0.106 |
|  | **Nr (V) *vs.* Cr (V)** | **Day 14** | **26.9%** | **17.9%** | **-0.048** | **38.1%** | **99.4%** | **0.797** | **0.012** |
|  | Cr (P) *vs.* CrB (P) | Day 1 | 25.0% | 11.3% | 0.051 | 32.6% | 97.6% | 0.421 | 0.092 |
|  | Cr (P) *vs.* CrB (P) | Day 8 | 26.0% | 19.0% | 0.068 | 30.8% | 95.8% | -0.416 | 1.000 |
|  | **Cr (P) *vs.* CrB (P)** | **Day 14** | **21.9%** | **17.9%** | **0.153** | **30.7%** | **97.9%** | **0.751** | **0.002** |
|  | Cr (V) *vs.* CrB (V) | Day 14 | 30.9% | 17.3% | 0.040 | 43.4% | 98.3% | 0.555 | 0.062 |

**Abbreviations:** Nr, Normal group; Cr, CCA group; CrB, CCA with bile group; P, Peripheral plasma; V, Portal vein plasma. The *p*-values for all models were calculated using permutation tests (n=500).

**Table S3**	Relative abundance of the top 20 family-level microbiota taxa at day 14

| **Family** | **Relative abundance (%)** | | | |
| --- | --- | --- | --- | --- |
|  | **Cr** | **CrB** | **Ct** | **Nr** |
| *Peptostreptococcaceae* | 40.35 | 19.68 | 35.72 | 43.76 |
| *Lactobacillaceae* | 38.30 | 60.54 | 45.74 | 27.33 |
| *Clostridiaceae* | 16.09 | 4.77 | 3.31 | 20.86 |
| *Lachnospiraceae* | 1.31 | 8.32 | 4.00 | 1.69 |
| *Ruminococcaceae* | 1.11 | 1.00 | 1.43 | 1.14 |
| *Oscillospiraceae* | 0.56 | 0.66 | 0.86 | 0.46 |
| Unknown | 0.52 | 0.65 | 5.33 | 1.94 |
| *Enterococcaceae* | 0.50 | 0.29 | 0.05 | 0.46 |
| *Erysipelotrichaceae* | 0.36 | 0.22 | 0.13 | 0.58 |
| *Streptococcaceae* | 0.22 | 0.57 | 0.19 | 0.03 |
| *Monoglobaceae* | 0.14 | 0.19 | 0.51 | 0.39 |
| Others | 0.11 | 0.19 | 0.19 | 0.12 |
| *Muribaculaceae* | 0.11 | 0.20 | 0.39 | 0.13 |
| *Eubacterium* | 0.07 | 0.09 | 0.24 | 0.17 |
| *Enterobacteriaceae* | 0.07 | 1.30 | 0.00 | 0.01 |
| *Christensenellaceae* | 0.06 | 0.07 | 0.27 | 0.26 |
| *Butyricicoccaceae* | 0.05 | 1.04 | 0.20 | 0.06 |
| *Eggerthellaceae* | 0.03 | 0.03 | 0.07 | 0.05 |
| *Anaerovoracaceae* | 0.03 | 0.07 | 0.07 | 0.07 |
| *Bacteroidaceae* | 0.02 | 0.12 | 0.00 | 0.00 |
| *Aerococcaceae* | 0.00 | 0.00 | 1.30 | 0.49 |

**Table S4**	Relative abundance of the top 20 genus-level microbiota taxa at day 14

| **Genus** | **Relative abundance (%)** | | | |
| --- | --- | --- | --- | --- |
|  | **Cr** | **CrB** | **Ct** | **Nr** |
| *Romboutsia* | 40.10 | 19.55 | 35.51 | 43.52 |
| *Lactobacillus* | 27.09 | 30.44 | 36.30 | 23.60 |
| *Clostridium* | 16.08 | 4.76 | 3.28 | 20.85 |
| *Ligilactobacillus* | 6.03 | 20.20 | 4.38 | 0.96 |
| *Limosilactobacillus* | 5.18 | 9.88 | 5.06 | 2.77 |
| Unknown | 1.51 | 1.73 | 6.83 | 3.01 |
| *Ruminococcus* | 0.93 | 1.85 | 1.10 | 1.11 |
| *Lachnospiraceae* | 0.72 | 0.34 | 0.68 | 0.61 |
| Others | 0.61 | 1.23 | 1.43 | 0.95 |
| *Enterococcus* | 0.50 | 0.29 | 0.05 | 0.46 |
| *Turicibacter* | 0.33 | 0.10 | 0.07 | 0.47 |
| *Streptococcus* | 0.22 | 0.57 | 0.19 | 0.03 |
| *Monoglobus* | 0.14 | 0.19 | 0.51 | 0.39 |
| *Blautia* | 0.13 | 5.39 | 1.89 | 0.27 |
| *UCG_005* | 0.11 | 0.11 | 0.57 | 0.11 |
| *Eubacterium* | 0.10 | 0.18 | 0.33 | 0.13 |
| *Lachnoclostridium* | 0.10 | 0.23 | 0.35 | 0.22 |
| *Escherichia_Shigella* | 0.07 | 1.30 | 0.00 | 0.01 |
| *UCG_008* | 0.04 | 0.99 | 0.16 | 0.04 |
| *Fusicatenibacter* | 0.00 | 0.68 | 0.00 | 0.01 |
| *Aerococcus* | 0.00 | 0.00 | 1.30 | 0.49 |
